# Health Tract, No. 146

**Published:** 1865

**Authors:** 


### HEALTH TRACT, No. 146.

### DROWNING.

As multitudes go a bathing during the heats of summer, and even the very best swimmers are liable to be drowned, perhaps more liable than others, from their very fearlessness, it is a proper precaution for every individual to be familiar with the means of resuscitation. The London physicians advise,

1. To send instantly for a medical man, and while he is coming, place the patient in the open air, unless the weather is very cold; expose the face and chest especially to the breeze.

2. *To clear the throat.*—Place the patient gently face downward, with one wrist under the forehead, in which position all fluids will escape by the mouth, and the tongue itself will fall forward, leaving the entrance into the windpipe free. Assist this operation by wiping and cleansing the mouth. If there be breathing, wait and watch; if not, or if it fail, then,

3. *To excite respiration.*—Turn the patient well and instantly on the side, and,

4. Excite the nostrils with snuff, hartshorn, volatile salts, or the throat with a feather, etc., and dash cold water on the face, previously rubbed warm. If there be no success, lose not a moment, but instantly begin,

5. *To imitate respiration.*—Replace the patient on his face, raising and supporting the chest well on a folded coat or other article of dress.

6. Turn the body very gently on the side and a little beyond, and then briskly on the face, alternately; repeating these measures deliberately, efficiently, and perseveringly, about fifteen times in the minute, or every four seconds, occasionally varying the side. [By placing the patient on the chest, its cavity is compressed by the weight of the body, and expiration takes place; when turned on the side, this pressure is removed, and inspiration occurs.]

7. On each occasion that the body is replaced on the face, make uniform but efficient pressure, with brisk movement on the back, between and below the shoulder-blades or bones, on each side, removing the pressure immediately before turning the body on the side.

8. After respiration has been restored, promote the warmth of the body by the application of hot flannels, bottles or bladders of hot water, heated bricks, etc., to the stomach, the arm-pits, between the thighs, and to the soles of the feet, to induce circulation and warmth.

9. During the whole time do not cease to rub the limbs upward, with firm, grasping pressure, and with energy, using handkerchiefs, flannels, etc.

10. Let the limbs be thus warmed and dried, and then clothed, the bystanders supplying the requisite garments.

*Cautions.*—1. Send quickly for medical assistance and for dry clothing. 2. Avoid all rough usage and turning the body on the back. 3. Under no circumstances hold up the body by the feet; 4. Nor roll the body on casks; 5. Nor rub the body with salts or spirits; 6. Nor inject tobacco smoke or infusion of tobacco. 7. Avoid the continuous warm bath. 8. Be particularly careful, in every case, to prevent persons crowding around the body.

*General Observations.*—On the restoration of life, a teaspoonful of warm water should be given; and then, if the power of swallowing has returned, small quantities of wine or brandy and water, warm, or coffee. The patient should be kept in bed, and a disposition to sleep encouraged. The treatment recommended should be persevered in for a considerable time, as it is an erroneous opinion that persons are irrecoverable because life does not soon make its appearance, cases having been successfully treated after persevering several hours.

In endeavoring to rescue a drowning person, *take him by the arm from behind*, between the elbow and the shoulder. A good swimmer can, by "treading water," catch both arms thus, and keep the person from going under for an hour, the very struggles of the victim aiding in buoying him up, for his feet then are mainly engaged, and he also, to that extent, "treads water." If a drowning person is seized anywhere else, he is pretty sure to clutch with a death-grip, and both perish.

Any one can remain for hours in water, whether he can swim or not, by clasping his hands behind him, throwing himself on his back, so as to allow only his nose to be out of the water; a very little presence of mind, force of will, and confidence, will enable any one to assume this position.

